# Impact of health systems reform on COVID-19 control in Sierra Leone: a case study

**DOI:** 10.1186/s41182-023-00521-z

**Published:** 2023-05-17

**Authors:** Tracey Elizabeth Claire Jones-Konneh, Angella Isata Kaikai, Ibrahim Borbor Bah, Daisuke Nonaka, Rei Takeuchi, Jun Kobayashi

**Affiliations:** 1 Department of Global Health, Graduate School of Health Sciences, University of the Ryukyus, 207 Uehara, Nishihara-Cho, Nakagami-Gun, Okinawa, 903-0215 Japan; 2 Pharmaceutical Society, Freetown, Sierra Leone; 3 Ministry of Health and Sanitation, Freetown, Sierra Leone; 4 Sierra Leone Psychiatric Hospital, University of Sierra Leone Teaching Hospital Complex, Freetown, Sierra Leone; 5 District Health Management Team, Pujehun, Sierra Leone; 6 Graduate School of Public Health, International University of Health and Welfare 4-3, Koudunomori, Narita, Chiba, 286-8686 Japan

**Keywords:** Case study, COVID-19, Ebola, Health system reform, Infectious disease control, Sierra Leone

## Abstract

**Background:**

There are various impacts of COVID-19 on health systems of the world. The health systems of low- and middle-income countries are less developed. Therefore, they have greater tendencies to experience challenges and vulnerabilities in COVID-19 control compared to high-income countries. It is important to contain the spread of the virus, and likewise strengthen the capacity of health systems in order for the response to be effective and swift. The experience from 2014 to 2016 Ebola outbreak in Sierra Leone served as preparation for COVID-19 outbreak. The aim of this study is to determine how control of COVID-19 outbreak in Sierra Leone was enhanced by the lessons learned from 2014 to 2016 Ebola outbreak, and health systems reform.

**Methods:**

We used data from a qualitative case study conducted in four districts in Sierra Leone through key informant interviews, focus group discussions, document, and archive record reviews. A total of 32 key informant interviews and 14 focus group discussions were conducted. A thematic analysis was used to analyze the data, and all transcripts were coded and analyzed with the aid of ATLAS.ti 9 software program.

**Results:**

The six themes obtained were composed of categories that connect with each other and with codes to form networks. The analysis of the responses demonstrated that “Multisectoral Leadership and Cooperation”, “Government Collaboration among International Partners”, and “Awareness in the Community” were among the key interventions used during the control of 2014–2016 Ebola virus disease outbreak, which were applied in the control of COVID-19. An infectious disease outbreak control model was proposed based on the results obtained from the analysis of the lessons learned during the Ebola virus disease outbreak, and health systems reform.

**Conclusions:**

“Multisectoral Leadership and Cooperation”, “Government Collaboration among International Partners” and “Awareness in the Community” are key strategies that enhanced the control of the COVID-19 outbreak in Sierra Leone. It is recommended that they are implemented in controlling COVID-19 pandemic or any other infectious disease outbreak. The proposed model can be used in controlling infectious disease outbreaks, especially in low- and middle-income countries. Further research is needed to validate the usefulness of these interventions in overcoming an infectious disease outbreak.

## Background

Health systems have been placed under immense pressure by Corona virus disease 2019 (COVID-19) pandemic which has produced sudden substantial impacts around the world. In developed and developing countries, there are shortages, and challenges in supply and access of certain products due to the pressure placed by the pandemic on public procurement systems [1]. A survey conducted in the five World Health Organization (WHO) regions showed that all national health systems are being tested by the pandemic, and in almost every country essential health service delivery and utilization has been affected [2]. In every society, for the most vulnerable populations, there is an impact that is large enough to affect availability of, and access to high-quality services even though the extent of the impact at the population level is generally partial (5–50%) [2]. All around the world, international and national health institutions have been active since the start of the outbreak, although there has been an extensive overbearing effect of COVID-19 on these institutions [3]. Developed countries are struggling with efforts to contain the spread and withstand the destructive effects of the pandemic even though they have technologically advanced and well-equipped systems. Developing countries are also struggling in their efforts to contain the coronavirus pandemic. Many developing countries have a challenge with lack of intensive care unit beds which threatens their abilities to respond to increase in COVID-19 cases [3]. In order to achieve universal health coverage for all, the health systems in developing countries should be strengthened, and responsive and resilient especially during an outbreak [4]. Sierra Leone which is not an exception is located on the west coast of Africa, bordered on the north and east by Guinea, on the south by Liberia, and on the west by the Atlantic Ocean.

In Africa, there is a great challenge with susceptibility of health systems. Almost all the countries in Africa have weak health care systems, and as a result there was a concern about the impact of the COVID-19 pandemic in the region even though the pandemic started late and progressed slowly. In Sub-Saharan Africa (SSA), the health systems are threatened by a lot of challenges that the COVID-19 pandemic has aggravated [5]. Some of these challenges are inadequate human resources and availability of essential medicines, insufficient financing through low budgetary allocation, and poor leadership and management [5].

Sierra Leone was fast to respond to the COVID-19 outbreak threats. Based on the previous Ebola response models, strategies such as ambulance services, preparedness plans, policy, coordination, and command and control structures were quickly activated. COVID-19 Emergency Response Centres were set up at the national and district level [6]. There was reactivation of social mobilization and community-based action groups which were important in the Ebola epidemic response. Hospital spaces with infrastructure and staff were converted to safe treatment and isolation centers in order to quickly extend COVID-19 treatment beds. COVID-19 treatment centers were not located at Non-Governmental Organization (NGO) facilities but were rather located at government hospitals [6].

Sierra Leone was seriously affected by Ebola Virus Disease (EVD) outbreak in 2014–2016. The national health systems did not have the scope needed to respond to an outbreak of that nature or provide the required life-saving health services, and therefore that outbreak drew attention to, and worsen the weaknesses and vulnerabilities in the health system [7]. The end of the EVD outbreak was declared on March 17, 2016 [8]. This outbreak had a notable effect on the weak health systems and played a part in decreasing the availability of human and physical resources for health [9]. The health workforce density was 2.2 per 10,000 population which was below WHO’s recommendation of 4.45 per 1000 [10]. A total of 221 healthcare workers (HCWs) died of EVD from the start of the outbreak on May 25, 2014 unto November 7, 2015 [11]. The understaffed healthcare system was further weakened, adding extra burden on the HCWs which led to increased exhaustion and fatal mistakes. When health systems are fragile, a sudden demand on them can cause morbidity and mortality from infectious diseases to increase significantly. Inadequate education on infection, prevention, and control (IPC) was one of the reasons for the initial failure of the Ebola outbreak control [12]. Thus, trainings were organized for HCWs in order to decrease the infection rate. The first case of COVID-19 outbreak was recorded about four years after the Ebola outbreak, on March 31, 2020. A well supported health system will help to control the impact of COVID-19 pandemic and maintain essential health services.

The 2014–2016 Ebola outbreak experience helped Sierra Leone to prepare for the COVID-19 outbreak. This outbreak was the largest Ebola outbreak in history [13] and as a result Sierra Leone had a first-hand knowledge of the extraordinary impacts of a virus outbreak. In most African countries there are shortages of human resources for health (HRH) which was amplified by the occurrence of EVD in Sierra Leone [14]. Some important lessons in fighting infectious diseases such as spread of information through radio jingles, community engagement, partnership and collaboration with religious leaders were learned from the outbreak [15]. Risk perception increased among Sierra Leoneans because of their EVD experience and the news of the massive impact of COVID-19 in high-income countries (HICs).

Several lessons learned from the EVD response can be implemented in the COVID-19 response even though there are differences in their modes of transmission and the strategies used to flatten the curve of infection [16]. Strategies for an effective COVID-19 response also need a diversity of public health measures including robust surveillance, isolation and treatment of cases to limit or prevent transmission of the disease [17]. The lessons learned from social responses to EVD in Sierra Leone provided a useful starting point for the COVID-19 response even though these responses needed to be revised to address the challenges of COVID-19 [18]. There was an assumption that Sierra Leone was at a disadvantage in dealing with epidemic diseases due to the fact that the technical capacities of its health system are remarkably weak. The experience of EVD challenged this assumption by showing that local social knowledge is as important as scientific and technological capacity [18]. EVD as is COVID-19 was a new disease with no effective treatments but as it was initially assumed, the weak local health system was not the obstacle. The focus of EVD control was on preventing the spread of the disease which was done by developing a constructive system for identifying and isolating cases, and tracing and quarantining contacts. This system needed human resources and social knowledge which Sierra Leoneans were acquainted with [18].

The extent to which EVD cases can be tracked or isolated using local social knowledge was opposed to the British experience of COVID-19 in which the role of human resources was belittled and the power of new technology was considered the unavoidable solution to the epidemic challenge [18]. A smart phone application was used to develop a system for COVID-19 contact tracing in which the close social contacts of the infected case will be recorded automatically and advised to self-isolate. However, this system proved to be ineffective and it was abandoned.

The aim of this research is to determine how the control of COVID-19 outbreak in Sierra Leone was enhanced by the lessons learned from the 2014–2016 Ebola outbreak, and health system reform. Using qualitative methods, we intend to investigate how the lessons learned during the 2014–2016 Ebola outbreak were applied to COVID-19 control. Also to find out if any reform of the health systems took place after the Ebola outbreak, and how it helped the control of COVID-19 outbreak.In this study our desire is to involve a total of 104 individuals from four districts (Western Area Urban, Port Loko, Koinadugu and Pujehun) in Sierra Leone, during the period of June 2021 to May 2022, who witnessed both the 2014-2016 Ebola outbreak and COVID-19 outbreak. Our findings will contribute to the improvement of the existing model for the control of infectious disease outbreaks. Application of the results obtained from this study will be helpful for preparedness, response, and control of infectious disease outbreaks.

## Methods

### Study sites

**Fig. 1:**
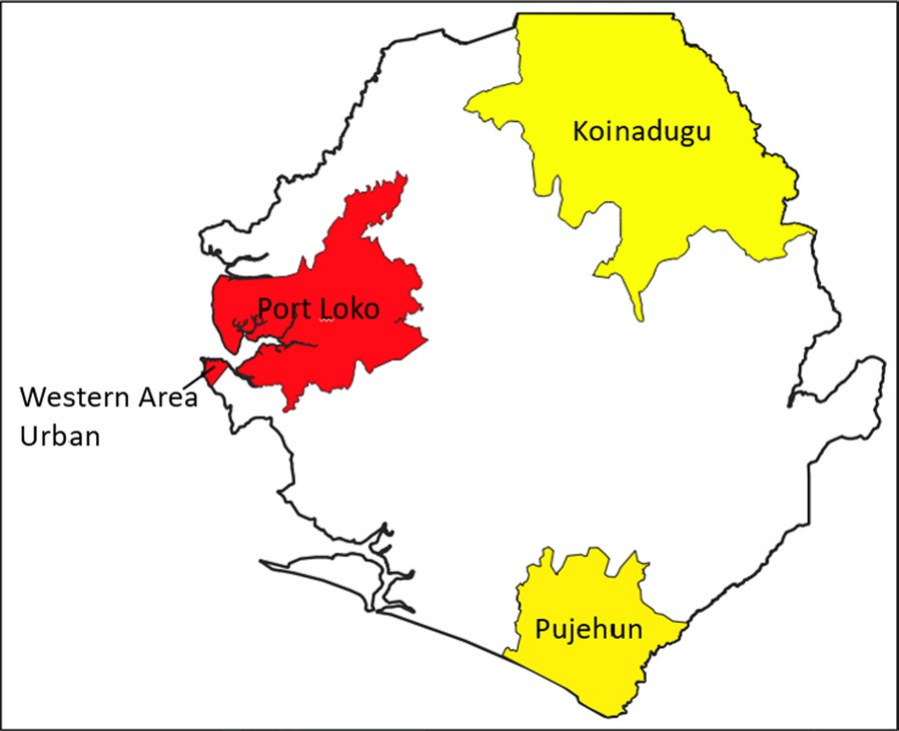
Study sites in Sierra Leone

Four districts (Western Area Urban, Port Loko, Koinadugu and Pujehun; Fig. 1) were chosen based on the incidence of EVD and COVID-19 cases. Koinadugu (EVD = 409,372; COVID-19 = 49) and Pujehun (EVD = 346,461; COVID-19 = 50) had lower numbers of both EVD and COVID-19 cases, whereas Western Area Urban (EVD = 1,055,964; COVID-19 = 4618) and Port Loko (EVD = 615,376; COVID-19 = 231) had higher numbers of cases. Six government hospitals that had a treatment or isolation center during the EVD outbreak and COVID-19 pandemic were selected. Three hospitals were chosen in Western Area Urban because each one has a specialty (referral hospital for surgical and medical cases, maternal hospital, and children’s hospital) and one hospital each was chosen from Port Loko, Koinadugu, and Pujehun, because they are general hospitals.

### Study participants

Participants were selected using purposive sampling. Respondents were selected if they experienced the 2014–2016 EVD outbreak, had roles and responsibilities on the control of EVD and COVID-19 outbreak, treatment of EVD and COVID-19 patients, community health, and planning of health system reform. They included representatives from the ministry of health and sanitation (MoHS), international non-governmental organizations (INGOs), community leaders and community members. Two HCWs refused to participate in the Key informant interviews (KIIs). It was intended that for each focus group discussions (FGDs) involving HCWs, five healthcare workers (two doctors, two nurses and one pharmacy professional) would be randomly selected from each district hospital at the study sites, but due to the limited human resources especially in the rural area, doctors were replaced with community health officers (CHOs). The FGDs for community leaders and community members also included five individuals each.

### Data collection

Key informant interviews, focus group discussions, and document and archive record reviews were conducted. Data were collected from June 2021 to May 2022. A total of 32 KIIs were conducted with governmental and non-governmental stakeholders (Table 1).

**Table 1:** Number of study participants by type of respondents and data collection method

No.	Types of respondents	Key informant interviews	Focus group discussions	Total participants
1	National level stakeholders	8	–	8
2	International non-governmental organizations	2	–	2
3	Health facility workers	22	30	52
4	Community leaders	–	15	15
5	Community members	–	25	25

All KIIs except one was held in Krio (a local language) which is spoken by approximately 97% of the population [19], the others were held in English. Fourteen FGDs were conducted with HCWs, community leaders and community members. The discussions with HCWs were held in English, while those with the community leaders and members were held in Krio. The sessions were conducted using the Zoom application, and recorded by researchers after gaining informed consent from the participants. The KII questions were divided into two groups: questions related to governmental and non-governmental stakeholders (Table 2).

**Table 2:** Key informant interview and focus group discussion questions

No.	Governmental stakeholder’s interview questions	International non-governmental stakeholder’s interview questions	Focus group discussion questions
1	How was the health system transformed after the Ebola outbreak?	How different is the role of your organization during EVD from that during COVID-19?	How are the ways in which Ebola was controlled different or similar to those used for controlling COVID-19?
2	How well improved is the health system since the end of the 2014–2016 Ebola outbreak?	How did your organization help in health system reform after the EVD outbreak?	How are the lessons learned from controlling Ebola used to control COVID-19?
3	How are the challenges of the control of COVID-19 overcome?		How was the health system transformed after the Ebola outbreak?
4	Why do you think the infection and death rate of COVID-19 patients is smaller compared to Ebola virus disease?		How are the challenges of the control of COVID-19 overcome?
5	How are the lessons learned from controlling Ebola applied to COVID-19?		Why do you think the infection and death rate of COVID-19 patients is smaller compared to Ebola virus disease?
6			How have the building blocks of health systems been improved?

Documents related to policies on health care and control measures for COVID-19 were reviewed. The Google search engine was used to search for documents online with a combination of the search terms: health care policies, COVID-19, control measures, infection prevention and control policy, Sierra Leone, and Free Health Care Initiative. The reviewed documents (Table 3) that contained policy development processes, strategic plans and responsible parties provided additional context to the findings from the KIIs and FGDs.

**Table 3:** Policy documents reviewed

No.	Reviewed documents	Date of documentation	Responsible organization
1	National Health Sector Strategic Plan 2017–2021	September, 2017	Ministry of Health and Sanitation
2	Human Resources for Health Policy 2017–2021	2016	Ministry of Health and Sanitation
3	Free Healthcare Services for Pregnant and Lactating Women and Young Children in Sierra Leone	November, 2009	Ministry of Health and Sanitation
4	National Health Promotion Strategy of Sierra Leone (2017–2021)	December, 2016	Ministry of Health and Sanitation
5	Sierra Leone National Health Information System Strategic Plan (2017–2021)	2016	Ministry of Health and Sanitation
6	Sierra Leone Announces New Covid Restrictions as Infection Cases Rise Sharply	21 January, 2021	NACOVERC*
7	President Bio Announces New Measures to Curb Rising Covid Cases and Deaths in Sierra Leone	1 July, 2021	Office of the President
8	National Infection Prevention & Control Action Plan (2016–19)	July, 2015	Ministry of Health and Sanitation
9	National Infection Prevention and Control Policy	November, 2015	Ministry of Health and Sanitation
10	Policy For Community Health Workers in Sierra Leone	June, 2012	Ministry of Health and Sanitation
11	Statement by His Excellency, Dr. Julius Maada Bio, President of The Republic of Sierra Leone on The Three-Day Lockdown—30 April 2020	30 April, 2020	Office of the President

*National COVID-19 Emergency Response Center (NACOVERC)

Archive evidence, such as records showing donated items from local and INGOs to hospitals within the period of September 29, 2020 to December 21, 2021 were collected. Web pages related to health systems reform and control of the COVID-19 outbreak were searched.

### Data analysis

The data collected were analyzed using a thematic analysis. KIIs and FGDs recordings were transcribed and translated into English where necessary. All transcripts were coded and analyzed with the aid of ATLAS.ti version 9.0.24, a qualitative data analysis software. The results were triangulated using the document and archive record review. An infectious disease outbreak control model was proposed based on the results**.**

## Results

Six themes emerged: “Multisectoral Leadership and Cooperation”, “Preparedness and Response to an Outbreak”, “Government Collaboration among International Partners”, “Decentralization of Resources”, “Awareness in the Community”, and “Availability of Resources for Health Improvement”. Each theme, category, and sub-category (Table 4) are all essentially connected when controlling an outbreak.

**Table 4:** Themes, categories, and sub-categories

Number	Themes (6)	Number of Responses (n%)	Categories (14)	Sub-categories (6)
Theme 1	Multisectoral Leadership and Cooperation	10.76%	1. Competent and qualified leadership 2. Multisectoral approach	Leadership Healthcare workers Finance Health service Information Health commodities
Theme 2	Preparedness and Response to an Outbreak	33.97%	1. Training and raising awareness on an outbreak 2. Public education on outbreaks 3. Prompt and robust measures to overcome the outbreak 4. Experiences from an outbreak helps in the preparation of other outbreaks	
Theme 3	Government Collaboration among International Partners	8.23%	1. Collaboration with international partners to fight the disease 2. Support from international partners	
Theme 4	Decentralization of Resources	11.18%	1. Implementation of strategies across the country 2. Proper monitoring of limited resources	
Theme 5	Awareness in the Community	6.54%	1. Adequate and specific message for education 2. Community engagement	
Theme 6	Availability of Resources for Health Improvement	29.32%	1. Building of infrastructures 2. Strengthening Infection, prevention, and control	

### Theme 1. Multisectoral leadership and cooperation

This theme is composed of two categories (“Competent and Qualified Leadership” and “Multisectoral Approach”), which form a network between other categories or codes.

### Competent and qualified leadership

The government set up a COVID-19 Emergency Response Center at the national level (NaCOVERC) and at the district level (DiCOVERC) to coordinate the COVID-19 outbreak. It is a replication of the body that was set up during the Ebola outbreak.

…when COVID-19 came, the structures used to put Ebola under control were reactivated, the national emergency operation center for Ebola has now been termed NaCOVERC at the national level and DiCOVERC at the district level. (P6, Western Area)

…NaCOVERC is the strategic body, they put policies in place and ensure that these policies are being implemented; and they are the main controlling body of the outbreak. (P1, Port Loko)

Qualified personnel are involved in various sectors and those who had experience from the Ebola outbreak were brought on board.

…we placed people at places where we know they are comfortable, based on their experience. If we know that during Ebola you worked at the triage; you will work at the triage. If we know that during the Ebola you worked at the treatment center, we will place you at the isolation unit or the treatment center, we do not take chances. (P1, Kabala)

### Multisectoral approach

Fighting an outbreak involves more than the Ministry of Health. During the Ebola outbreak, the Ministry of Defense was in charge of quarantine homes, in order to enforce the law.

...the ministry alone is not handling COVID-19, there are other support ministries and partners that are giving support for COVID-19. The Ministry of Defense is supporting to make sure they enforce the law of compliance and even the supervision of supplies to see that people do not misuse supplies that are given for COVID-19. (P39, Western Area)

...the Ministry of Finance, Ministry of Water Resources, the Ministry of Roads, because there are hard to reach areas you need their support. We are working with other line ministries to ensure that we are able to manage and bring the pandemic under control. (P8, Western Area)

### Theme 2. Preparedness and response to an outbreak

Four categories are involved when preparing and responding to an outbreak: “training and raising awareness about an outbreak”; “public education on outbreaks”; “prompt and robust measures to overcome the outbreak”; and “experiences from an outbreak helps in the preparation for other outbreaks”.

### Training and raising awareness about an outbreak

Whenever an EVD outbreak occurs, it is expected to reemerge. Therefore, training of HCWs during and after the EVD outbreak helps in the preparation for any other outbreak.

...HCWs were trained, including community health workers (CHWs) that go into the community, and if they see any of those diseases, they identify them and the response will be prompt. (P4, Kabala)

### Public education on outbreaks

A communication link was set up to help the population with information about the outbreak, and to communicate any information about any case.

...district health management team (DHMT) has a social mobilization group that goes into the communities, they educate people about health, about the causes of COVID-19. That has contributed a lot to raising awareness about the pandemic, and we have radio discussions with the DHMT. They will go to the radio station, discuss a topic with the community, and they encourage the community to call and ask questions or give their concerns. (P3, Port Loko)

The population is educated in a local language that can be understood. The social mobilization team from the community goes around the community to sensitize the people about COVID-19.

...social mobilization is very important and strong because every district has a pillar. They are using all possible ways to ensure that the information gets to every citizen. They send out messages in different languages. (P4, Kabala)

### Prompt and robust measures to overcome the outbreak

A prompt response helped to control the spread of the disease. It was a lesson learned from the Ebola outbreak since a delayed response led to the spread of disease.

...We learned an important lesson that when you have an outbreak, you should respond immediately. Do not wait for people or money, otherwise we would have waited like we waited during EVD for three months trying to look to other people to do something, and then the disease will spread; however, this time we were very proactive. (P2, Western Area)

### Experiences from an outbreak help in the preparation for other outbreaks

They built on the existing structures available, and the strategies implemented during the EVD outbreak.

...the last thing that happened before the end of the EVD which was very successful was community engagement. We had community mobilizers, printed out T-shirts for them with the slogan "community get the power for stop Ebola", and those community neighbors watch; community engagement actually helped to stop EVD. We based it on the existing strategy to fight COVID-19, and that is why it is working. (P, INGO2)

### Theme 3. Government collaboration among international partners

“Collaboration with International Partners to Fight the Disease”, and “Support from International Partners” are categories under this theme. They connect with other categories and codes.

### Collaboration with international partners to fight the disease

The government and HCWs had no knowledge on the control of EVD, therefore they had to work with international partners to control the outbreak. This was also implemented during the COVID-19 outbreak, and funding to the health sector was increased.

...We have partners’ meetings, and discuss the priorities in the health sector and how to combat the challenges. After Ebola we realized there was a need for the government to implement the health sector strategic plan, because that is a guiding document and that is what a lot of partners are now doing. (P8, Western Area)

...The WHO is supporting the ministry to do national health accounts, and recently worked with the office of the vice president to launch the national platform for the delivery of Universal Health Coverage. (P, INGO1)

### Support from international partners

International partners have been supporting the health sector in various ways to strengthen it and help overcome the COVID-19 outbreak.

...we are implementing directly; fuel for the transportation of samples, and for the daily running of the hospital or DHMT. We donated IPC items to peripheral health units (PHUs), trained HCWs on IPC measures. We also support the in-charge’s meetings where we disclose COVID-19 key messages. (P, INGO2)

...our major challenge in the implementation phase was funding, but the World Bank supported it, we had support from the Center for Disease Control (CDC), WHO regional office through the country office, FCDO (Foreign Commonwealth and Development Office). (P4, Western Area)

### Theme 4. Decentralization of resources

Decentralization of resources consists of two categories, “implementation of strategies across the country”, and “proper monitoring of limited resources”, which connects with other categories and codes.

### Implementation of strategies across the country

The implementation of strategies was not limited to capital cities alone, chiefdoms were also included.

...surveillance officers were only at the district level, they go to the field to do surveillance and come back, but now, if a particular district, for example, has ten chiefdoms, each of these ten chiefdoms have surveillance officers. They have the responsibility to see that they constantly monitor all the health facilities within that particular chiefdom. (P6, Western Area)

More HCWs are being absorbed into the health system, and are being distributed to various districts. There are training of HCWs who also add up to the number of the health workforce.

...government has embarked on recruiting a lot of health workers including doctors, nurses, and even support staff. The frontline workers have increased in terms of number, because there is an increase in the number of doctors specifically for the outpatient service and even nurses that attend to doctors in giving support for outpatients. (P24, Western Area)

...Many people have been trained, we have the FETP (field epidemiology training program), almost every district has these personnel and they serve as the frontliners whenever we have an outbreak in any part of the country. (P1, Kabala)

### Proper monitoring of limited resources

Acquiring logistics from other countries is difficult during a pandemic. Therefore, proper management of resources is needed since there might be limited resources.

...During Ebola one of the challenges was that a lot of partners came in with their money to support but those moneys were fragmented. It was a big challenge to trace where moneys were going, but at the middle of COVID-19, the ministry brought everybody on board to ensure that resources are pulled, so that there will be efficient use of those resources. (P8, Western Area)

### Theme 5. Awareness in the community

Awareness in the community involves “Adequate and Specific Message for Education”, and “Community Engagement” which are connected with other categories and codes.

### Adequate and specific message for education

The right information about the disease given to the community is simplified so as to enhance proper understanding of the message sent.

...We have a community definition for particular diseases. The clinical definitions are different from the community definitions of these conditions. Once CHWs have been trained and sensitized on the community definition of infectious diseases, they can easily pick them up. The definitions are pictural definitions, some of them cannot read effectively so the pictural definition can help them. (P6, Western Area)

Community mobilizers disseminate information to their communities. The messages are translated to their local languages in their communities so that the people can better understand.

...community mobilizers engage communities, give them information to keep them safe. They give them information during their community interphase meetings where you have the community mobilizer, community members themselves, and HCWs. (P, INGO2)

...there are populations that you have to talk in their local language, which means that you have to translate all the information from English to Krio, to all the different local languages that we have in the country, and this is being done. (P36, Western Area)

### Community engagement

The community takes ownership of the disease outbreak in their community. They implement by-laws, carry out sensitization to create awareness among community members.

...Community involvement is very important, and that was a very good tool that was used during Ebola. We used the community health volunteers who are located in various villages to bring information. (P2, Port Loko)

...During the community engagement, the communities will come up with action plans. If you are found guilty of breaking that law, you pay a fine, and in the Sierra Leonean context nobody wants to pay a fine, nobody wants to disobey the Chief. (P, INGO2)

CHWs who are within the community help the HCWs go to areas that are difficult to reach.

...Within the community we have the CHWs who are volunteers. The trained health personnel cannot be stationed in every area of the village or the country. The CHWs are trained in basic skills in terms of how to identify unusual conditions in the community. (P6, Western Area)

### Theme 6. Availability of resources for health improvement

“Building of Infrastructures”, and “Strengthening Infection, Prevention and Control” are categories that are connected to other categories and codes.

### Building of infrastructures

Infectious disease wards were built; hospitals, laboratories were renovated to help improve the health system.

...After the Ebola outbreak, the government equipped some hospitals with improved laboratory services, delivery services, as well as ensured that we have a lot of practitioners and consultants, so that in the case that emerging diseases occur we may be in a better position to defeat them. (P1, Port Loko)

Some medicines are available free of charge at the free health care pharmacy, but those that are not available can be bought from the private public partnership (PPP) or cost recovery pharmacy at a reasonable cost.

...government sent free health care drugs to various hospitals, but the ones that are unavailable we get them from the cost recovery pharmacy. There are other cases coming into the hospital who are not beneficiaries of the free health care system, so they go to the cost recovery to get the essential drugs needed for their treatment. (P2, Port Loko)

...PPP was brought in because the government thought there was a need for access when they could not provide it. The free health care could not provide all of those essential medications, so the patient went to the PPP pharmacy to buy these medicines at a minimal cost in comparison to other retail pharmacies. (P26, Western Area)

There was only one virology laboratory in the country, but that has been improved as more laboratories have been established. There are other systems that have been established that will help in the response of any outbreak.

...After the Ebola outbreak there was a need to reform the health system, especially towards responding to public health emergencies. The Directorate of Health Security and Emergencies, which deals with public health emergencies, disease surveillance, IPC, preparedness, One Health, Antimicrobial Resistance, was set up. (P3, Western Area)

### Strengthening infection, prevention, and control

A specific program was set up for IPC, and training on IPC was organized to strengthen the IPC in hospitals.

...the government ensured active IPC trainings or IPC practices in most of these hospitals. Most of the staff were trained in IPC, and also most of the hospitals were advised to ensure that IPC is paramount, and it's a must that people go through the IPC system. (P1, Port Loko)

## Discussion

This study showed that the lessons learned from the 2014–2016 Ebola outbreak and health systems reform played a great role in the control of the COVID-19 outbreak in Sierra Leone. “Multisectoral Leadership and Cooperation”, “Government Collaboration among International Partners” and “Awareness in the Community” were identified as findings to improve the existing Incident Management System (IMS) model which is used as the main approach in the management of WHO’s response to emergencies [20]. The main roles of IMS are finance and administration, health operations and technical expertise, information and planning, leadership, operations support and logistics, and partner coordination [20]. The findings of the present study are not limited to emergencies, they also apply to infectious disease outbreaks.

In the present study, competent and qualified leadership was identified as an important element of “Multisectoral Leadership and Cooperation”. The same structures that were used to control the EVD outbreak were reactivated during the COVID-19 outbreak. Although EVD and COVID-19 differ in their mode of transmission and pathogenesis, many facets of preparedness and the response to outbreaks of these two diseases overlap, and therefore key lessons learned from the response to the EVD outbreak are applicable to the response to COVID-19 [17]. Lessons learned from a previous infectious disease outbreak can be applied to future outbreaks.

Similar experiences were suggested in the following previous studies. Although there were few exceptions, the COVID-19 response in Morocco was led by reactivating the same structures that were used for coordinating the influenza A (H1N1) 2009 pandemic response [21]. The strong leadership from the Ugandan President and Ministry of Health applied in coordinating the COVID-19 national response was said to be responsible for the initial success of the COVID-19 pandemic control in Uganda. Uganda’s long experience to successfully control many previous epidemics such as HIV/AIDs in the 1980s, measles in the 1990s, hepatitis B in the 2000s, Ebola in 2000, 2017, and 2018, and Marburg in 2018, is believed to be the reason for this model of response [22]. In Singapore, the approach to the COVID-19 outbreak control has been evaluated as effective due to the fact that the infection rates have been kept low. One important factor that Singapore's success has been attributed to is the lessons learned from the experience obtained from an outbreak of hand–foot–mouth disease in 2000, severe acute respiratory syndrome (SARS) in 2003, and avian influenza H1N1 in 2009 [23].

The multisectoral approach, which this study describes, is suggested to be essential for countering emerging infectious diseases. According to a study done in Ethiopia, the multisectoral approach entails conscious cooperation amidst several stakeholder groups (e.g., government, civil society, and private sector) and sectors (e.g., health, environment, and economy) to accomplish a policy outcome together [24]. The Ministry of Health of the Federal government of Ethiopia (FMOH) carried out all the preparations needed to address the COVID-19 pandemic. Regional governments and city administrations were brought on board to fight the disease in collaboration with the federal government, civic organizations, multi-lateral and bilateral organizations, NGOs and GOs, universities and research institutions, religious institutions, and the public at large [24]. Likewise, the South Nations Nationalities and Peoples Region (SNNPR) government set up taskforces in response to COVID-19 pandemic [24].

In South Africa, MSA which is a principal occupant of the national response to HIV has evolved over time. The 1994 National AIDS Plan emphasized the incorporation of non-state actors, as well as formation of multisectoral structures to organize implementation of the country’s response to HIV at national and provincial level [25]. The concept ‘MSA’ was formally referred to and included in the National Strategic Plan (NSP) (2007–2011) which recognized that government alone will not be able to develop and execute a comprehensive response to HIV and AIDS, emphasizing the need to include non-government actors in the HIV policy process.

The MSA is amongst the key principles that frames the NSP 2012–2016, and the NSP 2017–2022. It is anticipated that through multisector collaboration, sectors such as government, civil society and the private sector in South Africa will work together, including pooling resources, knowledge, and expertise to tackle the complex social and structural drivers of the epidemic of HIV and AIDS. Collaboration of sectors occurs through AIDS Council structures that have been established at national, provincial, and local levels, mandated to work towards a common goal—a South Africa free from the burden of HIV—specified in the National Strategic Plan (NSP) on HIV and AIDS [25]. The South African National Department of Health used a multisectoral approach to contain and mitigate the spread of SARS-CoV-2 in South Africa, and government-wide decisions were made by the National COVID-19 Command Council [26].

In this study, “Collaboration with International Partners” is a relevant component in the fight against infectious diseases. The government of Sierra Leone, from their Ebola outbreak experience, figured out that collaboration is very important. Therefore, the National Health Sector Strategic Plan 2017–2021 (HSSP II) was executed during the COVID-19 outbreak to help in the collaboration with international partners. The heads of state of African Union countries made a declaration in Abuja, in April 2001, to allocate at least 15% of their annual budget to improve the health sector [27]. The government of Sierra Leone made an effort to increase this allocation, although it was not up to 15%, to help with the necessities of the health sector.

Experiences from previous outbreaks showed the importance of collaboration during a pandemic. Several countries agreed to work together to fight infectious diseases after the nineteenth century pandemics; and the COVID-19 pandemic has also revealed the value of international cooperation and collaboration [28]. Accepting rules and standards makes it possible to compare information, assist in the establishment of good practices, and encourages shared understanding and mutual trust [28].

Zambia experienced great international collaboration and support in fighting the pandemic. The Zambian government would have been overburdened by the pandemic if there was no local and international collaboration and support [29]. Therefore, Zambia has involved in continuous collaboration with other countries and organizations in order to contain COVID-19. In June, 2021, the European Union (EU) and the World Health Organization Representative Office for Malaysia, Brunei Darussalam, and Singapore, launched a three-year program to support the government of Malaysia in its COVID-19 response and preparedness for future pandemics [30]. The aim was to strengthen pandemic preparedness and to support Malaysia deal with this health emergency and its consequences.

“Support from International Partners” was seen in this study as a component of “Government Collaboration among International Partners”, which helps to overcome infectious disease outbreaks. This was also seen in 2020 when the European Union mobilized an emergency budget of EUR 86.5 million to help Ghana handle the consequences of the coronavirus pandemic; this gave Ghana the fiscal space to handle the pandemic and to continue financing basic public services, such as health care and education, strengthening its resilience [31]. In September 2020, Zambia received a donation of critical medical equipment from the United State government to fight the pandemic. Also, on the 20th October 2020, the government of the Republic of Korea in partnership with the World Health Organization donated COVID-19 test kits valued at $200,000 to strengthen the government’s national response to the pandemic [29].

Several international partners pledged their support to low-income countries in response to the COVID-19 pandemic. The World Bank on December 15, 2021 announced a USD 93 billion replenishment package from the International Development Association to assist low-income countries in response to the COVID-19 crisis and build a greener, more resilient, and inclusive future [32]. During the 2nd Global COVID-⁠19 Summit Commitments on May 12, 2022, France made a commitment of EUR 70 million to strengthen vaccine production capacity in developing countries, including Rwanda, Senegal, and South Africa [33]. Germany contributed especially in African countries 850 million euros as specific aid for vaccine logistics and strengthening of absorption capacities [33].

“Adequate and Specific Message for Education” was seen in this study as an important part of raising “Awareness in the Community”. Even though English is the official language of Sierra Leone, several local languages are spoken. Community mobilizers used the local languages of the communities to pass on information. Furthermore, various means of communication, such as radio and television, are utilized. This was also seen in South Africa where continuous communication with the population, and messages were disseminated through a variety of channels, including WhatsApp, radio, television, and the internet in all of the official South African languages [26].

The Democratic Republic of the Congo has more than 300 local languages, and five of them are national languages. Therefore, it was extremely important for community mobilizers to use the appropriate local language in their communities. It is vital during COVID-19 to use local languages for all risk communication and community engagement activities [16].

Community engagement is an important strategy used—according to this study—when carrying out interventions to raise awareness of an infectious disease outbreak in the community. It played a great role in the control of COVID-19 in Sierra Leone, wherein CHWs, social mobilizers and contact tracers were people within the community. Participation of the population in controlling outbreaks has always attested to be a principal element [34]. Health systems often depend on the mobilization of the population to assist in containing or mitigating health problems due to the risk of the spread of new or known diseases. Mobilization of the society has recently been used in several countries to control diseases such as influenza A (H1N1), Ebola, dengue, and Zika [34].

According to a study done in three remote and isolated First Nations communities of sub-arctic Ontario, Canada, community engagement process had several benefits, in addition to obtaining valuable community-based input [35]. It is relevant for pandemic plans to address local beliefs and values; therefore, it is crucial for citizens to play a part in pandemic planning as they can give invaluable insight into local perspectives. Also, it is specifically important to engage and address concerns expressed by disadvantaged populations, such as First Nations living in remote and isolated communities, as they encounter unique living conditions and are envisioned to be unfairly affected by a public health emergency [35].

The use of CHWs within the community screening and contact tracing strategy was recognized as a major strength during the first surge in South Africa [26]. Recommendations for the COVID-19 response based on lessons learned from the EVD outbreak in the Democratic Republic of the Congo showed that engagement with trusted community, political, and religious leaders suggest an essential gateway to the community [16].

**Fig. 2:**
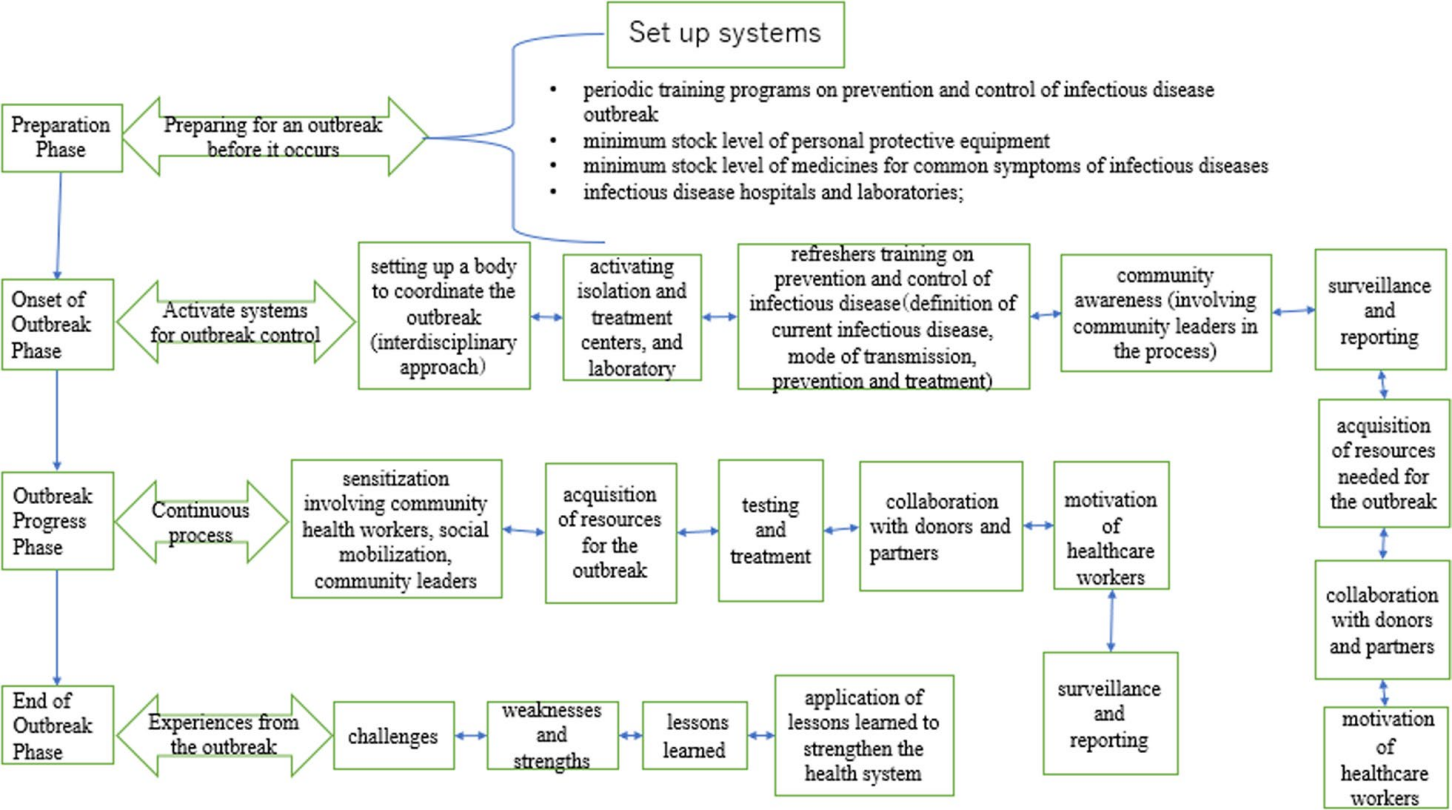
Proposed infectious disease outbreak control model

The model for infectious disease outbreak control was proposed based on an analysis of the results obtained from this study (Fig. 2). The “preparation phase” involves setting up systems such as training, storage of logistics, and building of infrastructure. At the “onset of outbreak phase”, activities such as activating emergency response centers, community awareness, surveillance, and reporting are implemented, among others. The “outbreak progress phase” involves a continuous process of sensitization, collaboration with donors, partners, and other stakeholders until the outbreak comes to an end. Finally, in the “end of outbreak phase” experiences from the outbreak that can be applied to improve the health system, and to future outbreaks, can be recognized. This can be used to complement the WHO’s Incident Management System, especially in the outbreak preparation phase.

## Conclusion

“Multisectoral Leadership and Cooperation”, “Government Collaboration among International Partners” and** “**Awareness in the Community” are key strategies that enhanced the control of the COVID-19 outbreak in Sierra Leone. It is recommended that they are implemented in controlling the COVID-19 pandemic or outbreaks of any other infectious disease. The comprehensive model can be used to help in the control of infectious disease outbreaks, especially in low- and middle-income countries. The covers the whole period from the start to the end of an outbreak, and also involves preparing for future outbreaks based on lessons learned from the previous outbreak. Further studies are needed to validate the interventions needed to overcome an infectious disease outbreak.

## Data Availability

Not applicable.

## Abbreviations

AIDs: Acquired immunodeficiency syndrome
CDC: Center for Disease Control
CHOs: Community health officers
CHWs: Community health workers
COVID-19: Corona virus disease 2019
DHMT: District Health Management Team
DiCOVERC: District COVID-19 Emergency Response Center
EVD: Ebola Virus Disease
FCDO: Foreign Commonwealth and Development Office
FETP: Field Epidemiology Training Program
FGDs: Focus group discussions
HCWs: Healthcare workers
HIV: Human immunodeficiency virus
HICs: High-income countries
HRH: Human resources for health
HSSP II: Health Sector Strategic Plan 2017–2021
IMS: Incident management system
INGOs: International non-governmental organizations
IPC: Infection, prevention and control
KIIs: Key informant interviews
MoHS: Ministry of Health and Sanitation
NaCOVERC: National COVID-19 emergency response center
NGO: Non-governmental organization
PHUs: Peripheral health units
PPP: Private public partnership
SARS: Severe acute respiratory syndrome
SSA: Sub-Saharan Africa
WHO: World Health Organization
