# Correction: Impact of health systems reform on COVID-19 control in Sierra Leone: a case study

**DOI:** 10.1186/s41182-023-00539-3

**Published:** 2023-08-24

**Authors:** Tracey Elizabeth Claire Jones-Konneh, Angella Isata Kaikai, Ibrahim Borbor Bah, Daisuke Nonaka, Rie Takeuchi, Jun Kobayashi

**Affiliations:** 1 Department of Global Health, Graduate School of Health Sciences, University of the Ryukyus, 207 Uehara, Nishihara-cho, Nakagami-gun, Okinawa 903-0215 Japan; 2 Pharmaceutical Society, Freetown, Sierra Leone; 3 Ministry of Health and Sanitation, Freetown, Sierra Leone; 4 Sierra Leone Psychiatric Hospital, University of Sierra Leone Teaching Hospital Complex, Freetown, Sierra Leone; 5 District Health Management Team, Pujehun, Sierra Leone; 6 Graduate School of Public Health, International University of Health and Welfare 4-3, Kouduno-mori, Narita, Chiba 286-8686 Japan

**Correction: Tropical Medicine and Health (2023) 51:28** 10.1186/s41182-023-00521-z

Following publication of the original article [1], the authors reported that the fifth author’s name needed to be corrected from “Rei Takeuchi” to “Rie Takeuchi”.

The correct author’s name has been provided in this Correction.

The original article [1] has been corrected.
